# Construction of a questionnaire based on the Health Action Process Approach for psycho-social cognitive determinants of parents in brushing children’s teeth in the Netherlands

**DOI:** 10.1371/journal.pone.0289337

**Published:** 2023-08-03

**Authors:** Karin Alexandra van Nes, L. Andries van der Ark, Cor van Loveren, Irene Helena Adriana Aartman

**Affiliations:** 1 Department of Paediatric Dentistry, Academic Centre for Dentistry (ACTA), University of Amsterdam and VU Amsterdam, Amsterdam, The Netherlands; 2 Research Institute of Child Development and Education, University of Amsterdam, Amsterdam, The Netherlands; 3 Department of Oral Public Health, ACTA, University of Amsterdam and VU Amsterdam, Amsterdam, The Netherlands; Shahid Beheshti University of Medical Sciences School of Dentistry, ISLAMIC REPUBLIC OF IRAN

## Abstract

**Background:**

The health action process approach (HAPA) model is promising to increase the frequency of brushing children’s teeth by parents to improve their children’s oral health. A validated HAPA questionnaire is needed as one of the measures of the effects of such an intervention.

**Objectives:**

The aim of this study was to evaluate whether our data, based on a translated and adopted version of the Health Action Process Approach (HAPA)-based questionnaire on dental flossing, supported the constructs of the HAPA model. If so, a next aim was to assess whether these constructs could be measured reliably.

**Methods:**

In this cross-sectional study, 269 questionnaires filled out in dental offices by parents of children 1–10 years old were analysed. Scale validation was performed according to the 6-step protocol of Dima, including Mokken scale analyses (MSA), graded response model (GRM), factor analyses and reliability measures. Pearson correlation coefficients were calculated to identify divergent validity and test-retest reliability.

**Results:**

MSA showed a unidimensional, medium total scale. Three items were removed based on this analysis. The total scale with the remaining 26 items did not fit the GRM. Factor analysis extracted five factors and two components for the total scale. The separate subscales, except the ‘intention’ construct, fitted the MSA and did not fit the GRM. The data fitted a seven-factor model better than a one-factor model. Reliability measures varied from acceptable to excellent, but were poor for ‘action control’. Test-retest reliability (r’s 0.60–0.83) was questionable to good.

**Conclusion:**

Our results did not fully support the constructs of the HAPA model. To support the HAPA constructs, modification to the subscales risk perceptions, intention, action planning, action control and self-reported behaviour are suggested. With these adjustments, the reliability and validity of the questionnaire could be significantly improved”.

## Introduction

In the Netherlands, at least 24% of five-year-old children suffer from cavitated dental caries [1], a condition that can have negative effects on the quality of life of children [2, 3]. To prevent dental caries, twice daily brushing with fluoridated toothpaste is the recommended oral health behaviour [4, 5]. As children lack the motoric skills to clean their teeth properly until they are ten years old, it is advised that parents brush their children’s teeth up until that age [6]. Hooley et al. [7] showed in their review that a lack of parental oral health knowledge is associated with an increased caries risk in children. Parents benefit from educational oral health interventions, but prevention programmes that focus on the family’s situation are more successful than programmes that focus on increasing parental knowledge of oral health [8]. In general, increasing a person’s knowledge on benefits or harms of specific behaviour does not necessarily lead to behaviour change because it usually neglects the ecological perspective, including the underlying mechanism of behaviour change [9]. To create and maintain positive oral health behaviour, insight in the psychosocial background of the parents is required, as well as support for parents in implementing healthy behaviour [10].

**Fig 1:**
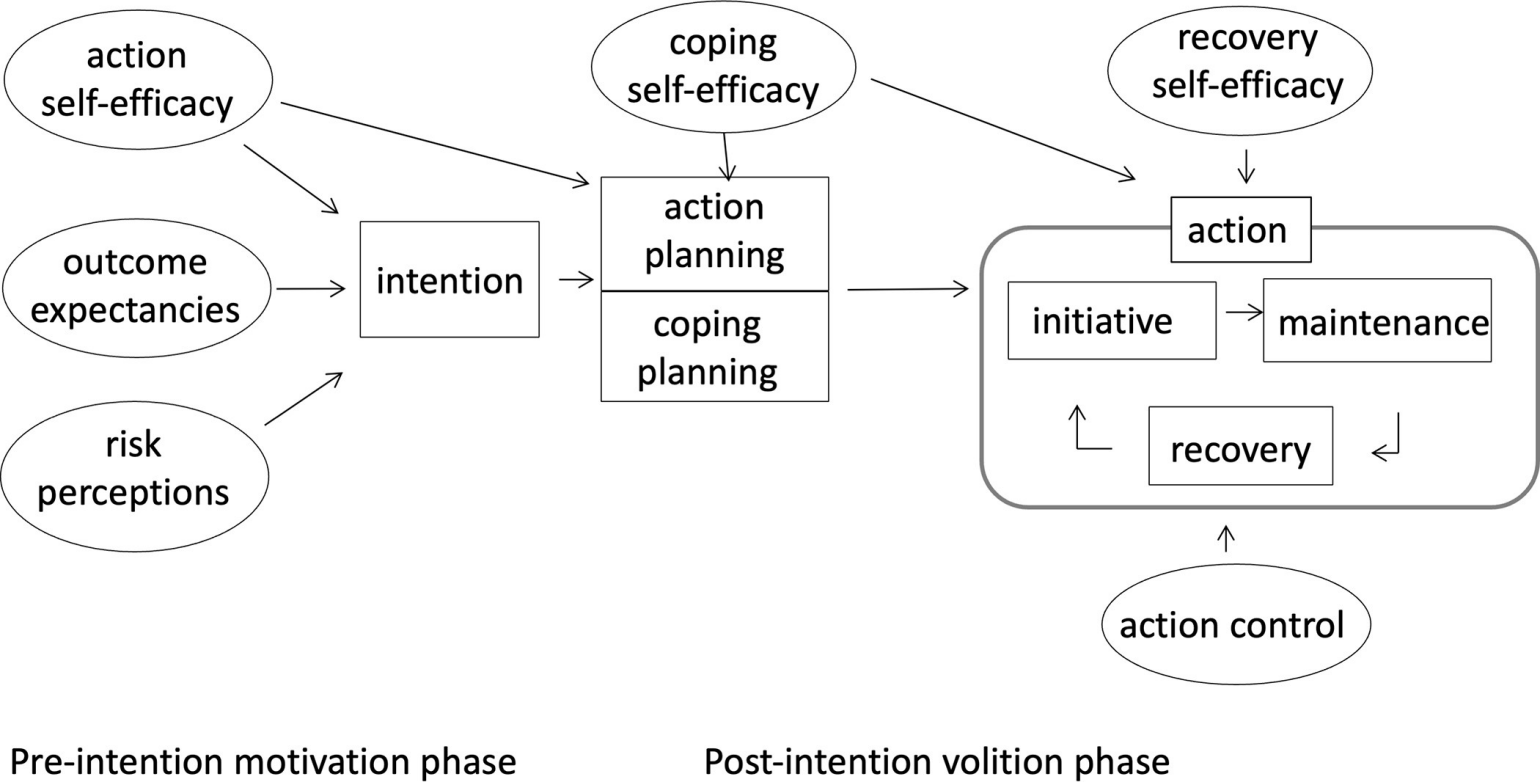
The HAPA model, adapted from Schwarzer [12].

Prevention programmes could become more effective when they are built on a theory of behaviour change, such as the Health Action Process Approach (HAPA) [11]. The HAPA incorporates the psychological process of intention formation into the actual change in behaviour. The model (Fig 1) is built on social cognitive constructs to bridge the gap between intention and actual behaviour change [12]. HAPA-based interventions have been successfully implemented in the promotion of a healthy diet [13], physical activity [14–16] and medication intake [17]. In oral health research, an intervention based on the HAPA has had a positive effect on flossing behaviour [18–20]. Evidently, it would be interesting to use the HAPA in improving tooth brushing in children. Until now, to the best of our knowledge, only one study [11] has been published on the mediating effects of planning, self-efficacy and action control on parental supervision for their children’s tooth brushing. The results suggest that the use of self-regulatory components might increase parental participation in behavioural change programmes to improve children’s oral health.

To study the effect of prevention programmes for parents in brushing children’s teeth based on the HAPA model, a validated HAPA-based questionnaire is necessary. Such a questionnaire does not yet exist. There is, however, an HAPA-based questionnaire for an intervention programme on dental flossing [21]. Although this HAPA-based questionnaire has been called a validated questionnaire [21], information on the item quality and factor structure could not be retrieved. This questionnaire on dental flossing was translated and adapted to a questionnaire for brushing children’s teeth. The aim of this study was to evaluate whether our data based on this questionnaire support the constructs of the HAPA model. If so, a next aim is to assess whether these constructs can be measured reliably.

## Materials and methods

### Study design and sample selection

A cross-sectional survey was administered to parents of children of 1- to10-years of age. Parents were approached by a research assistant to participate in the research and to fill out the questionnaire in the waiting room of dental practices in the Netherlands. These practices were the clinic of the Paediatric Department of the Academic Centre for Dentistry Amsterdam (ACTA), one referral practice for paediatric dental care, five general dental practices, and two offices of a large centre for dental care for school-aged children (4- to 18-years of age) that works in collaboration with elementary schools.

Parents could be included if they had a child between 1- to 10-years of age, if they were able to communicate in Dutch and if they had signed the informed consent form. Only one parent per child could participate, and parents could participate for one child only. The needed required sample size was calculated using criteria of Nunnally [23] for factor analysis, which required ten respondents per item of a questionnaire. Considering 29 items in the HAPA-based questionnaire for factor analysis, a sample size of 290 respondents would justify statistical analysis. For the test-retest reliability 48% of the parents were given a duplicate of the questionnaire and were requested to fill it out at home after two weeks and to return it by return-envelope.

### The questionnaire

For this research, a questionnaire was composed of 30 HAPA questions, nine locus of control questions, 16 socio-demographic variables and questions concerning oral health behaviour.

### HAPA subscales

This new questionnaire was based on the questionnaire of Gholami and Schwarzer on dental flossing [21] which contained 25 HAPA items that were categorised in eight constructs of the HAPA model (Table 1). Table 1 displays the definitions of the social cognitive constructs in the HAPA model. The model consists of two phases. First, in the motivational phase, intention is formed by socio-cognitive constructs, ‘risk perceptions’, ‘outcome expectancies’ and ‘action self-efficacy’. Subsequently, the volitional phase describes the role of ‘action planning’, ‘coping planning’, ‘coping self-efficacy’, ‘recovery self-efficacy’, and ‘action control’ (initiative, maintenance and recovery) on realising the behaviour. The model highlights the effect of self-efficacy in each stage of the behavioural change process. With the exception of the two open-end items about ‘oral health behaviour’, these original items could be answered on a 4-point Likert type scale ranging from very unlikely (1) to very likely (4) for the constructs ‘risk perceptions’ and ‘outcome expectancies’, and from not true at all (1) to definitely true (4) for the constructs ‘intention’, ‘action self-efficacy’, ‘coping self-efficacy’, ‘action planning’, ‘coping planning’ and ‘action control’.

**Table 1:** Subscale description [22].

Scale	Ab.	Description
Outcome expectancies	OE	Understanding of the contingencies between a person’s actions and subsequent outcomes
Risk perceptions	RP	Perceived severity of a health condition and personal vulnerability toward it
Action self-efficacy	aSE	Beliefs in one’s capabilities to exercise control over challenging demands and over one’s own functioning
Intention	INT	Motivation to alter the previous way of life and set goals for a different course of action
Coping self-efficacy	cSE	Optimistic beliefs about one’s capability to cope with barriers that arise during the period of behavioural maintenance
Action planning	AP	Planning to connecting the individual with good opportunities to act trough a task-facilitation strategy
Coping planning	CP	Protecting good intention from anticipated obstacles via a distraction-inhibiting strategy
Action control	AC	Self-regulatory strategy for promoting maintenance of an enacted behaviour through the continual monitoring and evaluation of a behaviour against a desired behavioural standard.

Ab. = Abbreviation

To adapt the questionnaire to tooth brushing of children aged 1 to 10 years by their parents, ‘dental flossing’ was replaced by ‘brushing my child’s teeth’. Consequently, the questionnaire was translated into Dutch using a forward-backward linguistic translation method [24], performed by two independent translators, a native Dutch speaker who speaks English fluently and a native English speaker who speaks Dutch fluently. Cultural and linguistic adaptations [25] were taken into account, making the questionnaire suitable for the target population. The translators received instructions from the first author [KvN] in advance to ensure uniformity in the translation process and contextual concept formation. Use of a dictionary was not recommended in order to prevent words being translated literally [26].

Once the questionnaire was back translated to English, it was compared with the original questionnaire by a panel. The panel consisted of six persons: one dental undergraduate student, two paediatric dentists, two non-paediatric dental researchers and one bilingual translator (other than the translators). The members of the panel had knowledge of or experience with treatment of children and communication with parents. Before the panel discussions, the members of the panel rated each question on comparability and interpretability using the rating sheet from Sperber 2014 [25]. The members of the panel indicated which constructs the question reflected. Also, in case of doubt or disagreement, they discussed and reformulated the questions. The adaptations are listed in S1 Table. As a result of the panel discussion, one double barrelled item of the construct ‘action planning’ was split into two items (items AP1 and AP2). In addition, five items specific for the target population were added: three for ‘risk perceptions’ [RP1, RP2, RP6], one for ‘coping self-efficacy’ [cSE2], and one for ‘coping planning’ [CP4]), whereas one open-end item was deleted *(How many times in last week did you floss*?*)*. Thus, the 25 original HAPA items were extended to 30 HAPA-based items to measure the nine constructs of the HAPA model: one open-ended item for the construct oral health behaviour: ‘*How many times a day in the past week did you brush your child’s teeth*’ and 29 4-point Likert type scale items to measure the subscales ‘intention’ (two items), ‘risk perceptions’ (six items), ‘outcome expectancies’ (three items), ‘action self-efficacy (three items), ‘coping self-efficacy’ (four items), ‘action planning’ (four items), ‘coping planning’ (four items) and ‘action control’ (three items) (Table 2). All Likert scales were modified into the answering options strongly disagree (1), disagree (2), agree (3) and strongly agree (4), which were considered to be more applicable than the original options. For each subscale, the respondent’s mean score was used as the measurement value. If a respondent had more than one missing item score in a certain subscale, the mean score was not computed, and the respondent’s measurement value was considered unavailable. Higher scores indicated a more positive cognition.

**Table 2:** Descriptive statistics. Response frequencies (*n*), mean* and standard deviations for 29 HAPA items per subscale before missing value imputation.

Scale	Item	Subscale/ Item stem	*N*	Item-frequency dist.				*M*	*SD*
				1	2	3	4		
OE		If I brush my child’s teeth on a daily basis…							
	OE1	. . .my child will feel good with beautiful teeth	268	3	29	164	72	3.14	0.63
	OE2	. . .my child will remain having healthy teeth	262	6	37	125	94	3.14	0.68
	OE3	…people in my community will see that my child is a clean person	263	20	94	115	34	2.63	0.80
RP		If I don’t brush my child’s teeth daily then…							
	RP1	. . .my child will be at risk for developing tooth decay	267	3	25	135	104	3.27	0.67
	RP2	. . .the new permanent teeth will be harmed	265	6	42	137	80	3.11	0.74
	RP3	. . .my child might lose his/her teeth too soon	267	9	41	162	55	2.99	0.70
	RP4	. . .my child might have bad breath	262	5	18	164	75	3.19	0.63
	RP5	. . .my child will be at risk for developing gum diseases	263	9	34	140	80	3.12	0.75
	RP6	. . .my chill will need braces in the future	267	102	139	20	6	1.74	0.69
aSE		I am confident that I immediately can start brushing my child’s teeth daily…							
	aSE1	. . .even if others do not brush their children’s teeth	263	5	21	142	95	3.24	0.67
	aSE2	. . .even if i have to force myself to do so	255	10	24	148	73	3.11	0.72
	aSE3	. . .even if it is time consuming	261	9	26	141	85	3.16	0.73
INT		I intend to brush my child’s teeth properly …							
	INT1	. . . once a day	259	25	55	117	62	2.84	0.89
	INT2	. . .at least twice a day	264	8	37	125	94	3.16	0.77
cSE		I am confident that I can continue daily brushing my child’s teeth…							
	cSE1	. . .even when I cannot see any positive changes immediately	264	3	14	147	100	3.31	0.62
	cSE2	. . .even when my child does not cooperate	264	5	29	133	97	3.23	0.71
	cSE3	. . .even when I am in a hurry	267	2	42	141	82	3.13	0.69
	cSE4	. . .even when it takes a long time to become part of my routine	261	6	19	146	90	3.23	0.67
AP		I have mad e concrete plan…							
	AP1	. . .how much time to spend with brushing my child’s teeth	266	10	76	129	51	2.83	0.77
	AP2	. . .how to brush my child’s teeth	269	7	70	137	55	2.89	0.75
	AP3	. . .how often to brush my child’s teeth	266	11	58	129	68	2.96	0.80
	AP4	…when and where to brush my child’s teeth	267	8	63	138	58	2.92	0.75
CP		To keep brushing my child’s teeth in difficult situations. I have made a concrete plan…							
	CP1	. . .in case something interferes with brushing my child’s teeth	263	23	114	92	34	2.52	0.83
	CP2	. . .in case I am in a hurry	262	17	111	97	37	2.59	0.80
	CP3	…in case my child has pain. Bleedings gums or tooth decay	266	22	107	105	32	2.55	0.81
	CP4	. . .in case my child does not cooperate	266	13	106	103	44	2.67	0.81
AC		During the past week…							
	AC1	. . .really tried to brush my child’s teeth daily	263	11	44	121	87	3.08	0.80
	AC2	. . .often had my intention of brushing my child’s teeth on my mind	258	15	66	116	61	2.87	0.83
	AC3	. . .consistently monitored how. When and how often I have brushed my child’s teeth	266	31	97	106	32	2.52	0.85

Note

*Mean scores after imputation of missing values (*n* = 269)

OE = outcome expectancies

RP = risk perceptions

aSE = action self-efficacy

INT = intention

cSE = coping self-efficacy

AP = action planning

CP = coping planning

AC = action control

Understanding of the questions were tested on four parents meeting the inclusion criteria. The graduate student who was working as a research assistant was present while parents completed the questionnaire to assist in understanding, interpretation and comprehension of the questions. The feedback from these parents resulted in minor revisions in sentence structure to enhance understanding of the questionnaire.

#### Locus of control

To assess the divergent validity of the HAPA subscales, an additional construct was measured. This construct was *locus of control*. For this purpose, the nine items selected and translated by Duijster et al. [27] from Lencová et al. [28] were used. Originally, these items were extracted from a validated questionnaire on oral health behaviour [29, 30]. In our questionnaire, we used nine items concerning tooth brushing with a four-point Likert answering scale to form the *locus of control* construct. All items were reverse-scored and were recoded before analysis. High scores on the locus of control items accounted for a more internal locus of control whereas low scores on the locus of control items accounted for a more external locus of control.

#### Additional variables

Subsequently, for discriminant validity, items were added to the questionnaire. The highest level of completed education of the mother of the child was used as an indicator for socio-economic position. The answering options ranged from ‘no education’ to ‘university level of education’. The answers were dichotomised into ‘low level of socio-economic position’ (no education, elementary school, lower level of secondary or further education) and ‘high level of socio-economic position’ (higher level of secondary or further education and university) [31]. Caries experience was measured by three closed questions used in previous research [32]: ‘*Did your child ever had a tooth extracted*?*’* (yes of no), ‘*Did your child ever had a tooth filled*?*’* (idem) and ‘*How would you describe the condition of your child’s teeth*?*’* (excellent, very good or good, fair or poor). Caries experience was dichotomised into ‘Caries free’ when teeth were neither extracted nor filled and the condition of the child’s teeth was described as excellent, very good or good by the parent. If either a tooth had been filled or extracted, or the condition of the child’s natural teeth was described as fair or poor, the caries experience was classified as ‘caries active’. The questions on demographic characteristics and oral health behaviour consisted among others of gender and age of the child, country of birth of the child, age of the mother, relationship to the child and marital status.

### Statistical analyses

#### Analyses following the scale validation protocol of Dima [33]

We performed scale validation according to the 6-step protocol of Dima [33].

*Step 1*. *Data control*. First, we checked the presence of impossible item scores. Then, we evaluated whether the assumption was tenable that non-observed item scores were missing completely at random using Little’s MCAR test [34]. If so, we used the two-way imputation analysis [35] replacing missing item scores with plausible item scores. *Outliers*. We used the number of Guttman errors to detect outlying response patterns [36]. As Guttman errors typically have a skewed distribution, response patterns having an outlier score greater than upper fence of the *adjusted boxplot* [37] were considered for removal. Subsequently, the presence of suspiciously small or large item-score means and standard deviations, and the presence of negative inter-item correlations was inspected.

*Step 2*. *Mokken scale analysis*. For the entire set of items and for each subscale separately, using a lower bound of *c* = 0.3, we used Mokken’s [38] automated item selection procedure to select the items and computed scalability coefficients *H* and *H*_*i*_*−*to investigate whether the items form a Mokken scale [38]. In addition, we investigated the assumptions of monotonicity using the method *manifest monotonicity* and invariant item ordering using *manifest invariant item ordering*. *Local independence* was inspected by checking for local conditional associations between items. Items that did not fit a Mokken scale were removed from further analyses.

*Step 3*. *Parametric item response theory analysis*. For the remaining items in the total scale and each subscale separately, we estimated a *graded response model* (GRM) [39] with a fixed discrimination parameter and a GRM with a free discrimination parameter for the total scale and each subscale. We used a benchmark of *χ*^2^<3.5 as an indicator of good fit for item pairs and item triples.

*Step 4*. *Factor analysis and item cluster analysis*. We conducted three types of factor clustering techniques on the remaining items in the total scale. First, *exploratory factor analysis*, in which both parallel analysis [40] and very simple structure (VSS) analysis [41] were used to determine the number of factors. Second, *hierarchical item cluster analysis* [42] was used to group items into clusters. Third, we performed *confirmatory factor analysis*, for which we used the following benchmarks as indicators of good fit (e.g., [43]: root mean square error of approximation (RMSEA) < 0.06, Tucker-Lewis index (TLI) and Comparative Fit Index (CFI) ≥ 0.95, standardized root mean square residual (SRMR) < 0.08, goodness-of-fit statistic (GFI) and the adjusted goodness-of-fit statistic (AGFI) ≥ 0.95. For both exploratory and confirmatory factor analysis, factor loadings greater than 0.30 or 0.40 on the hypothesized dimensions were considered as supporting the hypothesized dimension. We explored the fit of two confirmatory factor models. In one factor model, each factor corresponded to a HAPA scale, and in a second factor model, each factor corresponded to the Mokken scales obtained in Step 1.

*Step 5*. *Reliability analysis*. For the total score and for each subscale score separately, reliability was estimated using Cronbach’s alpha (α), Revelle’s beta (β), McDonald’s omega hierarchical (ω_h_) [44], and Guttman’s lambda-6 (λ_6_) [45]. Additionally to the protocol of Dima [33], for the total set of items, and for each subscale separately, we also calculated the corrected item-total correlations and considered a coefficient of at least 0.30 to be acceptable.

*Step 6*. *Subscale scores*. We presented means and standard deviations and ranges of the subscales. Pearson correlation coefficients were calculated between all HAPA subscales.

#### Test-retest reliability

The test-retest reliability of the subscales of the questionnaire was assessed by Pearson correlation coefficients. Coefficients ranging from 0.50–0.75 and from 0.75–0.90 were considered as respectively a moderate and good stability of the test over time [46].

#### Divergent validity

The *divergent validity* of the questionnaire was investigated by calculating Pearson correlation coefficients between locus of control and the HAPA subscales. Locus of control and self-efficacy are both related to parental oral health behaviour and their children’s oral health [47]. As locus of control represents a different socio-cognitive construct than risk perception and self-efficacy, it was hypothesised that locus of control correlates significantly, but weakly, with the HAPA subscales ‘risk perceptions’, ‘self-efficacy’ and ‘outcome expectancies’, but does not correlate with the other HAPA subscales.

#### Discriminant validity

It was hypothesised that parents of caries-free children have higher scores on the HAPA subscales ‘risk perceptions’, ‘action self-efficacy’, ‘outcome expectancies’ and ‘intention’ than parents of children who are categorised as caries active. Secondly, it was hypothesised that children with mothers with a high socio-economic position will have higher scores on the HAPA subscales ‘risk perceptions’, ‘action self-efficacy’, ‘outcome expectancies’ and ‘intention’ than mothers with a low socio-economic position. To determine if the questionnaire could discriminate between these groups the independent sample t-test was used.

Some of the analyses (e.g., missing value analysis, correlational analyses) were performed using IBM SPSS Statistics for Macintosh (Version 28). For all the other analyses the open source program R (version 1.4.1717, [48] was used, including packages mokken [49], psych [50], ltm [51], msm [52], polycor [53] and lavaan [54]. The R-code can be provided by the first author [KvN] upon request. A significance level of 1% was used.

### Ethics

The institutional review board of the Academic Centre for Dentistry Amsterdam (ACTA) approved the study and consent procedure (protocol number 2016 011). The board concluded that the Medical Research Involving Human Subjects Act (WMO) does not apply to this study and that the study is according to the ethical guidelines of ACTA. The consent procedure implied that the parents were informed verbally and in writing about the research, and signed the informed consent form before the study commenced.

## Results

A total of 290 questionnaires were collected. It was not recorded how many parents were approached and how many refused to participate. Therefore, a response rate cannot be calculated. From these questionnaires, some were excluded because it turned out that parents’ command of the language was insufficient to complete the questionnaire (*n* = 13) or because of more than 16 missing values on the HAPA items (*n* = 8). A total of 269 questionnaires remained for data analysis (doi.org/10.6084/m9.figshare.17294270.v1). Table 3 shows the characteristics of the sample.

**Table 3:** Sample characteristics of children and parents (*n* = 269).

Variables	Categories	*N*	Perc.
Dental practice	Paediatric department of ACTA	136	50.6
	Paediatric referral practice	61	22.7
	General practice	26	9.7
	Dental care centre for school-aged children	44	16.4
	NA	2	0.7
Child’s gender	Male	142	52.8
	Female	127	47.2
Child’s caries experience^a^	Free	68	25.3
	Active	197	73.2
	NA	4	1.5
Child’s country of birth	Netherlands	240	89.2
	European Union (Netherlands excluded)	2	0.8
	Other	7	2.7
	NA	20	7.4
Mother’s educational level^b^	Low	103	38.3
	High	157	58.4
	NA	9	3.3
Supervisor’s relation to child	Mother	191	71
	Father	76	28.3
	Other	2	0.07
Supervisor’s marital status^c^	With partner	211	78.6
	Single	51	19.1
	NA	3	2.2
Variables	*M*	*SD*	Range
Child’s age (n = 266)	6.6	2.13	1–10
Mother’s age (n = 265)	37.4	5.8	23–53
Brushing frequency^d^ (n = 245)	2.0	1.4	0–7

Note

Perc. = percentage

^a^ Caries free is applicable when parents reported no teeth were filled nor extracted and parents considered the teeth of their child to be excellent, very good or good.

^b^ High level of education: higher level of secondary or further education and university

^c^ Martial status with partner is applicable when married or living together with partner, while without partner is applicable for single, divorced/separated or widow/widower.

^d^ Recalculated brushing frequency per day, during last week.

### Analysis following the scale validation protocol of Dima [33]

#### Step 1. Data control

All response categories were represented in all HAPA items, indicating adequate spread across response categories (Table 2). Answers on the one open-end item on daily brushing frequency (item #34) ranged from 0 to 15. Most frequently provided responses were 0 (7.4%), 1 (17.8%), 2 (48.0%), 7 (3.7%) and 14 (3.3%). We considered some answers a result of misinterpretation of the question and divided all answers from seven and above by seven (e.g., 7 into 1, 14 into 2). After recalculation, the open-end item had a mean of 2.0 (*SD* = 1.4) (Table 3). Pearson correlations coefficients between items in the total scale ranged from 0.19 until 0.72; only one item (RP6) had negative inter-item correlations (S2 Table). Three HAPA items (cSE3, RP1 and AP2) had no missing values, eight items (aSE1, aSE2, aSE3, sSE4, CP1, CP2, AC2, INT1) had between 2% and 5.2% missing values, and the remaining items had less than 2% missing values. It was assumed that the unobserved scores were missing completely at random (Little’s MCAR test: *X*^2^ = 943.093, *df* =914, *p* = 0.245), and the missing item scores were replaced with plausible item scores. *Outliers*. No suspect response patterns were detected, and all data remained in the analysis.

#### Step 2. Mokken scale analysis

For the entire set of items, two items (RP6 and INT1) did not meet the criteria of a Mokken scale (Table 4). The remaining 27 items met the criteria of a Mokken scale. As *H* = 0.45 (*se* = 0.03), following Mokken’s benchmarks [38], the strength of the total scale could be labelled as ‘medium’. There were no significant violations of manifest monotonicity. For the remaining 27 items, there were several item pairs flagged as locally dependent item pairs. However, we found that the W-indices of the flagged item pairs were not outstanding compared to item pairs that were not flagged, and we decided to ignore the flags. The results on scalability, manifest monotonicity and local independence support the fit of Mokken’s monotone homogeneity model [55], which allows ordinal measurement using the total score. There were several violations of manifest invariant item ordering, indicating that the 27 items could not be ordered invariantly using the mean item score. When all subscales were analysed separately (Table 4), it appeared that item RP6 and the two items of the subscale ‘intention’ did not meet the criteria of a Mokken scale. Most item scalability coefficients (*H*_*i*_) were greater than 0.5. The subscale scalability coefficients (*H*) indicated a moderate scale for ‘action planning’ (>0.4) and strong scales for the other subscales (>0.5). No violations of manifest invariant item ordering or local independence were found for the subscales. Items RP6, INT1 and INT2 were removed, resulting in 26 items for further analyses.

**Table 4:** Measures for the entire set of items and the subscales for Mokken scale analysis.

Scale	Item	H_i_ total scale (se)	H_i_ subscale (se)	H index (se)	#vi MIIO subscale	*H* ^*T*^	CITR total scale	CITR Remaining set of items	CITR subscale
Total scale				0.401 (0.028)		0.29			
OE									
	OE1	0.308 (0.047)	0.598 (0.051)	0.588 (0.049)		0.55	0.46	0.46	0.71
	OE3	0.384 (0.041)	0.603 (0.054)				0.55	0.54	0.62
	OE2	0.352 (0.047)	0.563 (0.056)				0.53	0.53	0.66
RP (6 items)									
	RP6	0.020 (0.053)	-0.048 (0.075)	0.395 (0.040)		0.75	0.03		
	RP3	0.370 (0.043)	0.468 (0.044)				0.55		
	RP1	0.369 (0.048)	0.486 (0.043)				0.55		
	RP2	0.431 (0.041)	0.496 (0.040)				0.66		
	RP4	0.415 (0.048)	0.447 (0.051)				0.61		
	RP5	0.402 (0.047)	0.430 (0.056)				0.61		
RP (5items)									
	RP3		0.541 (0.051)	0.563 (0.042)		0.10		0.55	0.64
	RP1		0.603 (0.047)					0.56	0.72
	RP2		0.584 (0.044)					0.65	0.75
	RP4		0.547 (0.053)					0.61	0.66
	RP5		0.539 (0.055)					0.61	0.69
aSE									
	aSE2	0.378 (0.045)	0.565 (0.059)	0.590 (0.054)		0.06	0.56	0.55	0.66
	aSE1	0.441 (0.040)	0.635 (0.054)				0.66	0.66	0.75
	aSE3	0.439 (0.045)	0.571 (0.061)				0.66	0.66	0.69
INT									
	INT1	0.167 (0.053)	0.127 (0.088)	0.127 (0.088)			0.25		
	INT2	0.403 (0.043)	0.127 (0.088)				0.60		
cSE									
	cSE2	0.461 (0.035)	0.742 (0.039)	0.756 (0.036)		0.12	0.69	0.70	0.81
	cSE3	0.447 (0.036)	0.763 (0.038)				0.67	0.68	0.78
	cSE1	0.499 (0.036)	0.785 (0.040)				0.72	0.73	0.85
	cSE4	0.480 (0.038)	0.737 (0.046)				0.72	0.72	0.81
AP									
	AP4	0.425 (0.038)	0.584 (0.049)	0.658 (0.036)	2(0)	0.03	0.62	0.63	0.67
	AP1	0.482 (0.035)	0.699 (0.034)		1(0)		0.69	0.70	0.82
	AP3	0.479 (0.032)	0.697 (0.037)		1(0)		0.71	0.72	0.83
	AP2	0.456 (0.037)	0.653 (0.043)		0		0.66	0.66	0.78
CP									
	CP4	0.471 (0.034)	0.719 (0.040)	0.735 (0.032)		0.06	0.68	0.68	0.77
	CP1	0.465 (0.036)	0.773 (0.031)				0.68	0.68	0.88
	CP3	0.408 (0.040)	0.683 (0.043)				0.59	0.58	0.75
	CP2	0.486 (0.036)	0.764 (0.028)				0.70	0.70	0.88
AC									
	AC3	0.400 (0.042)	0.421 (0.067)	0.482 (0.052)		0.35	0.57	0.57	0.48
	AC2	0.356 (0.041)	0.511 (0.051)				0.53	0.51	0.66
	AC1	0.412 (0.044)	0.513 (0.053)				0.62	0.60	0.66

*H*_*i =*_ item scalability coefficient

*H =* scalability coefficient

#vi = number of violations with the significancy between parenthesis

MIIO = manifest invariant item ordering

*H*^*T*^ = item ordering coefficient

LI = local independence

CITR = item-total correlation corrected for item overlap and scale reliability

#### Step 3. Parametric item response theory analysis

Comparison of the two types of GRMs indicated that the GRM with a free discrimination parameter had a significantly better fit, both for the total scale and for the subscales ‘action planning’ and ‘action control’. However, for none of the subscales did the GRM with a free discrimination parameter fit the data well. The lack of fit may produce biased estimates of the latent trait values that are used for measurement.

#### Step 4. Factor analysis

**Fig 2:**
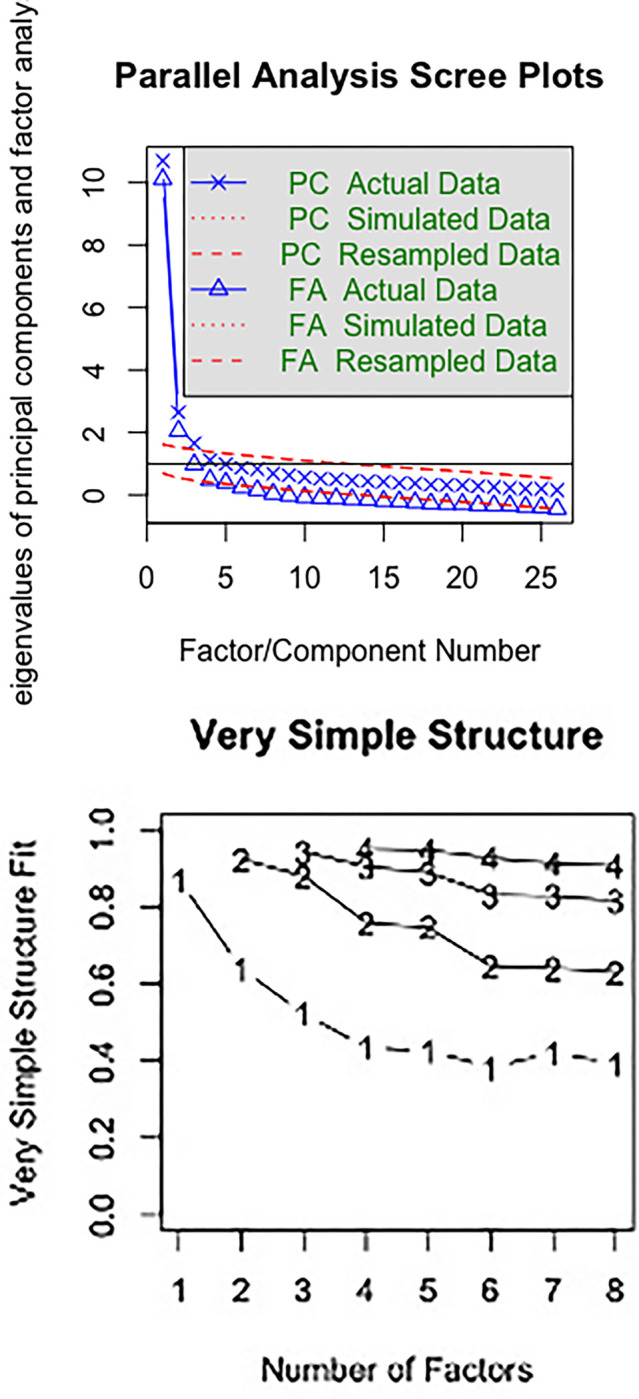
Plots of parallel analysis and very simple structure analysis of the remaining set of items (26 items).

The parallel analysis for the 26 remaining items proposed five factors and two components (Fig 2). Plots of parallel analysis and VSS analysis showed one main factor and a second minor factor (Fig 2). This suggested the total scale to be mostly unidimensional. Both parallel analysis and VSS analysis extracted one dominant factor and a secondary nuisance factor for subscale. Hierarchical cluster analysis identified first ‘outcome expectancies’. Then one cluster contained the items of ‘risk perceptions’, coping self-efficacy’, ‘action self-efficacy and ‘action control’. The other cluster contained the items of ‘action planning’, ‘coping planning’ and AC3. Except for the ‘action control’ items, further clustering distinguished the items in their anticipated components.

*Confirmatory factor analysis*. The fit indices of the remaining 26 items indicated a poor fit for the one-factor model. For the one-factor model, none of the fit indices met the requirement of the benchmarks (Table 5) indicating a poor fit. For the seven-factor model (all subscales except ‘intention’), RMSEA and SRMR met the requirements of the benchmarks, TLI and CFI did not, but were relatively close to their respective benchmarks, and GFI and AGFI did and were relatively low compared their benchmarks. Hence, there is no indication that the seven-factor model fitted well, nevertheless it fitted better than the one-factor model (Table 5).

**Table 5:** Measures for the remaining set of 26 items for confirmatory fit analysis.

Fit statistic	Model		Benchmark
	1-Factor model	7-Factor model	
χ^2^(*df*) RMSEA	1702834*(324) .13	558898*(278) .03	<.06
TLI	.64	.92	≥.95
CFI	.67	.93	≥.95
SMRA	.10	.06	<.08
GFI	.57	.86	≥.95
AGFI	.50	.83	≥.95

Note

*χ*^2^ (*df*) = chi square and degrees of freedom

RMSEA = root mean square error of approximation

TLI = Tucker-Lewis index

CFI = Comparative Fit Index

SMRA = Standardized root mean square residual

GFI = Goodness-of-fit statistic

AGFI = Adjusted goodness-of-fit statistic

#### Step 5. Reliability analyses

The reliability for the total scale was excellent (Table 6). For the subscales (Table 6), α ranged from 0.67 to 0.90, β ranged from 0.58 to 0.85, λ_6_ ranged from 0.53 to 0.88, and ω_h_ ranged from 0.03 to 0.90. The reliability for ‘action control’ would increase slightly (α = 0.69) after deletion of item AC3 from the scale. Nevertheless, we reasoned that as a minimum of three items per factor is needed [56] and a subscale of three items is preferred over a subscale of two items for a reliable subscale assessment [57]. Therefore, it was decided to maintain AC3 in the subscale action control. The corrected item-total correlations of the total scale ranged from 0.46–0.72 and from 048 to 0.88 for the subscales (Table 4).

**Table 6:** Reliability coefficients, mean and Pearson’s correlations between the subscales and locus of control.

Scale	Reliability coefficient				Test-retest stability (*n* = 30)	Divergent validity^a^
	α	β	ωh	λ_6_	*r*	*r*
OE	0.74	0.71	0.03			0.22
RP^b^	0.83	0.77	0.77	3.0	0.57	0.40
aSE	0.78	0.75	0.77	3.1	0.54	0.49
cSE	0.89	0.85	0.85	3.2	0.59	0.60
AP	0.87	0.83	0.82	3.2	0.59	0.40
CP	0.9	0.84	0.9	2.9	0.65	0.30
AC	0.67	0.58	0.13	2.6	0.71	0.29
Total^c^	0.94	0.83	0.7	2.8	0.64	

Note

^a^ Correlations of the HAPA subscales with locus of control

^b^ Risk perceptions without item RP6

^c^ remaining set of 26 items

α = Cronbach’s alpha

β = Revelle’s beta

λ_6 =_ Guttman’s lambda-6

ω_h =_ McDonald’s omega hierarchical

*r =* Pearson correlation coefficient. All correlations are significant at the 0.01 level (2-tailed)

#### Step 6. Total (sub)scale scores

The mean scores of the subscales ranged from 2.59 for *coping planning* to 3.22 for *coping self-efficacy*, and did not show any irregularities (Table 7). Pearson correlation coefficients between HAPA subscales indicated significant correlations between all HAPA subscales (Table 8). Correlation coefficients ranged from 0.35 to 0.74.

**Table 7:** Mean scores^a^ and standard deviations of the HAPA constructs for the total group (*n* = 269) and per caries experience group and per educational level of the mother and p values from independent-samples t-tests for the comparison of the groups.

	Total group		Caries free (*n*^*b*^≥ 66)	Caries active (*n*^*b*^≥ 193)		Low educational level of the mother (*n*^*b*^≥ 99)	High educational level of the mother (*n*^*b*^≥ 154)		
HAPA constructs	*N*	*Mean* (*SD*)	*Mean* (*SD*)	*Mean* (*SD*)	*p* value^c^	*Mean* (*SD*)		*Mean* (*SD*)	*p* value^c^
outcome expectancies	269	2.97 (0.58)	2.94 (0.63)	2.98 (0.56)	0.615	2.94 (0.62)		3.00 (0.55)	0.404
risk perceptions^d^	264	3.12 (0.54)	3.19 (0.51)	3.10 (0.56)	0.264	3.08 (0.59)		3.16 (0.50)	0.270
action self-efficacy	264	3.18 (0.60)	3.30 (0.56)	3.13 (0.61)	0.058	3.10 (0.67)		3.23 (0.55)	0.100
coping self-efficacy	265	3.22 (0.59)	3.37 (0.49)	3.17 (0.62)	0.018	3.17 (0.65)		3.25 (0.54)	0.347
action planning	269	2.90 (0.65)	2.98 (0.62)	2.88 (0.66)	0.267	2.82 (0.73)		2.96 (0.58)	0.102
coping planning	267	2.59 (0.72)	2.67 (0.71)	2.56 (0.72)	0.265	2.54 (0.76)		2.65 (0.69)	0.213
action control	265	2.82 (0.65)	2.88 (0.59)	2.81 (0.68)	0.427	2.75 (0.71)		2.88 (0.62)	0.110

^a^ Mean scores were computed with a maximum of one missing item score per subscale.

^b^ Variations in total number of cases are the results of case-by-case analysis. Therefore, the minimum number of cases for each subgroup is indicated.

^c^*p*-value of independent samples t-test

^d^ Risk perceptions without item RP6

**Table 8:** Pearson correlation coefficients between the HAPA subscales.

	Outcome expectancies	risk perceptions*	action self-efficacy	coping self-efficacy	action planning	coping planning
risk perceptions*	0.56					
action self-efficacy	0.35	0.57				
coping self-efficacy	0.39	0.66	0.70			
action planning	0.42	0.48	0.54	0.54		
coping planning	0.42	0.37	0.41	0.50	0.74	
action control	0.43	0.47	0.54	0.53	0.58	0.57

Note

Pairwise deletion of missing values

*Risk perceptions without item RP6

All *p* values < 0.001 (2-tailed)

### Test-retest reliability

Pearson correlation coefficients for the HAPA subscales between the test and retest ranged from 0.60–0.83 (*n* = 30), showing moderate to good stability (Table 6). The Pearson correlation coefficient of the recalculated open-end item on daily brushing frequencies showed a lower stability (*r* = 0.47, *p* = 0.021). The interval between the test and the retest ranged from two to four weeks with one outlier of two months.

### Divergent validity

Cronbach’s alpha of the locus of control subscale was α = 0.73. As predicted, the HAPA subscales ‘action self-efficacy’ (*r* = 0.49, *p*<0.001), ‘risk perceptions’ (*r* = 0.40, *p*<0.001) and ‘outcome expectancies’ (*r* = 0.22, *p*<0.001) correlated with locus of control (Table 6). The other HAPA subscales also showed positive correlations with the locus of control subscale (Table 6).

### Discriminant validity

Table 7 shows the mean scores per HAPA subscale for the high and low socio-economic position groups and for the caries free and caries active groups. For none of the HAPA subscales the mean test score differed significantly between the groups (Table 7).

## Discussion

This study investigated the HAPA-based questionnaire on brushing children’s teeth, which was adapted from the HAPA-based questionnaire on dental flossing. The aim of this study was to evaluate whether our data, based on this questionnaire, supported the constructs of the HAPA model. If so, a next aim was to assess whether these constructs can be measured reliably. Our first question was whether the data have the structure that is implied by the HAPA. Using more lenient models, we found indicators that the scale is approximately unidimensional. After deleting three non-fitting items using Mokken scale analysis we found no violations of the monotone homogeneity model, a non-parametric IRT model that assumes that a unidimensional latent variable explains the responses on the remaining 26 items. Also, exploratory factor analysis indicated that one dominant factor and several minor factors explained the structure of the data. However, we also found indicators that a unidimensional scale may be an oversimplification. More stringent models, such as the one-factor model in a confirmatory factor analysis did not fit the data well, whereas a seven-factor model (one factor for each HAPA scale measured) showed better fit. Other analyses were inconclusive. For example, the lack of fit of the graded response model may be due to the more stringent restrictions this model puts on the shape of the item characteristic functions, and a non-fitting graded response model does not necessarily imply a violation of unidimensionality. The question whether the subscales measure the same psychological construct or highly correlated separate psychological constructs remains unanswered. For many psychological constructs, test data show a single dominant dimension and several nuisance dimensions [58–60] and whether one interprets this as the presence of a single construct or several constructs that explain the test data is largely a matter of preference. Then, our sample of 269 was relatively small. There is a possibility, especially in exploratory methods that investigate the dimensionality of the data, such as Mokken scale analysis (Step 2) and exploratory factor analysis (Step 3), that the number of identified dimensions vary or are lower when using small samples compared to using a very large sample.The variety of reliability indices we used confirmed satisfactory reliability scores for all subscales. Only the Cronbach alpha for ‘action control’ was not satisfying. The construct ‘action control’ pertains to awareness of standards, that is, memorising the goals, self-monitoring, and self-regulation [61]. The coherence among the three action-control items is probably small resulting in low estimates of reliability. In addition, the HAPA items were stable over time.

The correlations between the HAPA constructs ‘outcome expectancies’, ‘risk perceptions’, ‘action self-efficacy’ on the one hand and ‘locus of control’ on the other were in accordance with our hypothesis. Furthermore, we found statistically significant correlations between the other HAPA constructs and ‘locus of control’. It is known that parental locus of control is related to caries experiences in children [62]. Therefore, it could be interesting to explore the potential moderating or mediating effect of locus of control on parental tooth brushing via HAPA constructs in future studies.

Our questionnaire could not discriminate between different socio-economic positions and caries experience groups. Results were non-significant using a 1% significance level and all HAPA subscales showed only small differences between groups. Our sample might have been too homogeneous and might have lacked diversity to discriminate. Firstly, the children of highly educated mothers were overrepresented in our sample. Possibly, parents of which the mothers are highly educated are more willing to participate in research. Secondly, our sample did include many caries active children (74.3%), since most children attended either a practice specializing in the treatment of children (67%) or a paediatric dental referral practice (22.7%). The prevalence of caries in 5-year-old children in the Netherland is 24% [1]. It was already shown by De Jong-Lenters et al. that in a referral practice the caries prevalence is higher than in a general practice [63]. It might be that more heterogeneity in caries activity is necessary to assess a relation with the HAPA-constructs. Thus, it cannot be excluded that the results from the present study would have been different when the sample contained more parents of children visiting the general dental practice. In a heterogeneous sample parents might have a different mind-set on brushing their children’s teeth than parents visiting the dental office for treatment of their child. On the other hand, the purpose of this validation study is to create a questionnaire that can be used in research with parents of high caries risk children, as these children could benefit most from a HAPA-based intervention to improve parent’s oral health behaviour regarding their children. Thirdly, the measure that we used for caries experience is subjective and therefore possibly biased. Nevertheless, in previous research, parents accurately assessed their children’s oral health status as children’s caries estimated by their parents were in concordance with the actual clinical situation [32, 64].

To summarize, although the scales of the questionnaire are reliable, our findings appear to be in conflict with the long-held support of the HAPA model [22]. It might be that the presence of the separate constructs of the HAPA model were never tested this thoroughly before, and that there is not enough evidence for the model. However, it cannot be excluded that some factors, partly related to our study, may also explain the results. To begin with, the final sample size was smaller than aimed. Today’s literature is inconclusive about the adequate sample for a factor analysis. Our sample size was based on the widely used item-to-respondent ratio by Nunnally [23]. Data collection stopped when 269 questionnaires were filled out, which resulted in a 1:9 item to respondent ratio. This ratio is considered to be sufficient for factor analysis [65]. Next, the constructs of the HAPA model are quite strongly correlated, which, as mentioned before, makes it difficult to unravel the subscales from the total scale. In addition, the high correlations between the subscales result in multicollinearity of the subscales that are used as predictors of ‘intention’ (‘action self-efficacy’, ‘outcome expectancies’ and ‘risk perceptions’), which may make it difficult to identify the effects (represented by the arrows in Fig 1). Furthermore, the parents of which the mother is relatively highly educated probably may know what actions and plans are good for their children’s oral health, but may not necessarily act accordingly. Since they filled out the questionnaires in the dental office, it may also be likely that parents gave socially desirable answers. Next, an important limitation was that children were in different stages of treatment in the practices at the time the parents filled out the questionnaire. That means that there was not necessarily an action plan discussed yet. Moreover, our results also indicated some weaknesses in the items of the questionnaire, which call for improvement and for analysing the adapted questionnaire again. For example, closer inspection of the items revealed that the ‘action planning’ item AP4 could be considered as a double-barrelled question, as it inquired where and when a parent is planning to brush the child’s teeth. For future research this item will be split into two questions in the ‘action planning’ component. Then, it might be that our methods were limited in determining the HAPA constructs and accompanying HAPA items. We believe that triangulation of research methods could contribute to the support of the HAPA model, for example by adding interviews with parents to distinguish their stages of behaviour change. Finally, by following the protocol of Dima, we performed a variety of analyses. Although each analysis has its individual characteristics, it may seem to be an overkill, even more so since they all showed us more or less the same pattern of results. That is, taking all analyses into account, our results complicate the modelling of the HAPA model.

In conclusion, our data do not fully support the constructs of the HAPA model. Our results suggest that the following changes might improve the questionnaire: removing items from the subscales risk perception and action control, adding items to the subscales intention and action control, as well as rewriting items of the subscales self-reported behaviour and action planning. We expect that an adapted version of the Health Action Process Approach questionnaire for parents in brushing their children’s teeth supports the constructs of the HAPA model and has significantly improved reliability and validity. Scale analysis is necessary in follow-up research to confirm our conclusion and to check whether the HAPA constructs can be measured in other samples.

## Supporting information

S1 Table Adaptation to the items. Item stem and items as originally formulated, after backwards translation and after panel discussion.
(DOCX)

S2 Table Inter-item correlation matrix of the entire set of items. (DOCX)
