# Supplementary material for: Construction of a questionnaire based on the Health Action Process Approach for psycho-social cognitive determinants of parents in brushing children’s teeth in the Netherlands
Source: PLoS One. 2023 Aug 3;18(8):e0289337. doi: 10.1371/journal.pone.0289337 (PMC10399854; doi:10.1371/journal.pone.0289337)
Supplement: S1 Table — Item stem and items as originally formulated, after backwards translation and after panel discussion. (DOCX) [file pone.0289337.s001.docx]

S1 Table. Adaptation to the items. Item stem and items as originally formulated, after backwards translation and after panel discussion.

| **Scale** | **Item** | **original** | **after backward translation** | **after panel discussion** |
| --- | --- | --- | --- | --- |
| OE |  | If I brush my child’s teeth frequently on a daily basis… | If I brush my child’s teeth daily, regularly….. | If I brush my child’s teeth on a daily basis… |
|  | OE1 | ...then my child will feel good with beautiful teeth all the time | . ..then my child will always feel happy with beautiful teeth | ...my child will feel good with beautiful teeth |
|  | OE2 | ...then my child will have healthy teeth for most of his life | ..then my child will have healthy teeth for the majority of their life | ...my child will remain having healthy teeth |
|  | OE3 | …then friends and parents will see that my child is a clean person | ...then my family and friends will think my child is a clean person | …people in my community will see that my child is a clean person |
| RP |  | If I don´t brush my child’s teeth frequently… | If I do not brush my child’s teeth regularly… | If I don’t brush my child’s teeth daily then… |
|  | RP1^a^ |  |  | ...my child will be at risk for developing tooth decay |
|  | RP2^a^ |  |  | ...the new permanent teeth will be harmed |
|  | RP3 | …then my child might lose some teeth prematurely | … then my child could lose some teeth prematurely | ...my child might lose his/her teeth too soon |
|  | RP4 | …then my child might have bad smell from his mouth | …than my child could develop bad breath. | ...my child might have bad breath |
|  | RP5 | …then my child will risk getting gum or periodontal diseases | …then my child is at risk of developing gum and periodontal illness | ...my child will be at risk for developing gum diseases |
|  | RP6^a^ |  |  | ...my chill will need braces in the future |
| aSE |  | I am confident that I can start brushing my child’s teeth immediately on a regular basis... | I am convinced that I can start brushing my child’s teeth on a regular basis… | I am confident that I immediately can start brushing my child’s teeth daily… |
|  | aSE1 | ...even if others do not brush their children’s teeth | …even if others do not brush the teeth of their children | ...even if others do not brush their children’s teeth |
|  | aSE2 | …even if I have to force myself to start brushing his teeth right now | …even if I have to force myself to start immediately with brushing his teeth | ...even if i have to force myself to do so |
|  | aSE3 | ...even if it is time consuming | …even if it is time consuming | ...even if it is time consuming |
| INT |  | I intend to brush my child’s teeth properly... | I have the intention to brush the teeth of my child very well… | I intend to brush my child’s teeth properly … |
|  | INT1 | …at least once a day | …at least once a day | ... once a day |
|  | INT2 | *Added after panel discussion* |  | ...at least twice a day |
| cSE |  | I am confident that I can frequently brush my child’s teeth regularly on a long-term basis… | I am convinced that I can brush my child’s teeth on a regular basis for a prolonged period of time… | I am confident that I can continue daily brushing my child’s teeth |
|  | cSE1 | ...even when I cannot see any positive changes immediately | …even if I do not observe any positive change immediately | ...even when I cannot see any positive changes immediately |
|  | cSE2^a^ |  |  | ...even when my child does not cooperate |
|  | cSE3 | …even when I am in a hurry | …even when I am in a hurry | ...even when I am in a hurry |
|  | cSE4 | …even when it takes a long time to become part of my daily routine | …even if it takes a while to make it part of my daily routine | ...even when it takes a long time to become part of my routine |
| AP |  | I have made a concrete and detailed plan regarding… | I have made a concrete and detailed plan that applies to… | I have mad e concrete plan… |
|  | AP1^b^ | …how to use a toothbrush with my child and how much time to spend brushing my child’s teeth | …how to use the toothbrush and how much time to spend on brushing my child’s teeth | ...how much time to spend with brushing my child’s teeth |
|  | AP2^b^ |  |  | ...how to brush my child’s teeth |
|  | AP3 | …how often to brush my child’s teeth | …how often I will brush my child’s teeth | ...how often to brush my child’s teeth |
|  | AP4 | …when and where to brush my child’s teeth (at which occasion) | …where and when to brush my child’s teeth (at which time) | …when and where to brush my child’s teeth |
| CP |  | To keep my brushing my child’s teeth habit in difficult situations, I have made concrete plan regarding… | I made a concrete plan to maintain my child’s teeth brushing habits in difficult situations concerning… | To keep brushing my child's teeth in difficult situations. I have made a concrete plan… |
|  | CP1 | ...what to do if something interferes with my goal of brushing my child’s teeth | …what to do when something prevents my goals of brushing my child’s teeth | ...in case something interferes with brushing my child’s teeth |
|  | CP2 | …what to do when I am in a hurry | …what to do when I am in a hurry | ...in case I am in a hurry |
|  | CP3 | …how to cope when my child has bleeding gums or pain | …how to respond when my child has bleeding gums or pain | …in case my child has pain. bleedings gums or tooth decay |
|  | CP4^a^ |  |  | ...in case my child does not cooperate |
| AC |  | During the last week… | During the past week… | During the past week… |
|  | AC1 | ...I have really tried hard to brush my child’s teeth frequently | …I have really tried to brush my child’s teeth often | ...really tried to brush my child’s teeth daily |
|  | AC2 | …I have often had my brushing my child’s teeth intentions on my mind | …I have had the intention to brush my child’s teeth in mind | ...often had my intention of brushing my child’s teeth on my mind |
|  | AC3 | …I have consistently monitored when, how often, and how to brush my child’s teeth | ….I have consistently monitored when, how often and how to brush my child’s teeth | ...consistently monitored how. when and how often I have brushed my child’s teeth |

*Note.*

OE = outcome expectancies

RP = risk perceptions

aSE = action self-efficacy

INT = intention

cSE = coping self-efficacy

AP = action planning

CP = coping planning

AC = action control

^a^ Item added after panel discussion

^b^ Item is split into two items
