# Supplementary material for: Construction of a questionnaire based on the Health Action Process Approach for psycho-social cognitive determinants of parents in brushing children’s teeth in the Netherlands
Source: PLoS One. 2023 Aug 3;18(8):e0289337. doi: 10.1371/journal.pone.0289337 (PMC10399854; doi:10.1371/journal.pone.0289337)
Supplement: S2 Table — (DOCX) [file pone.0289337.s002.docx]

S2 Table. Inter-item correlation matrix of the entire set of items

|  | OE1 | RP6 | aSE2 | AP4 | RP3 | cSE2 | AP1 | aSE1 | cSE3 | CP4 | AC3 | OE3 | RP1 | CP1 | AP3 | CP3 | AP2 | CP2 | OE2 | INT2 | AC2 | RP2 | cSE1 | cSE4 | RP4 | INT1 | AC1 | aSE3 | RP5 |
| --- | --- | --- | --- | --- | --- | --- | --- | --- | --- | --- | --- | --- | --- | --- | --- | --- | --- | --- | --- | --- | --- | --- | --- | --- | --- | --- | --- | --- | --- |
| OE1 |  |  |  |  |  |  |  |  |  |  |  |  |  |  |  |  |  |  |  |  |  |  |  |  |  |  |  |  |  |
| RP6 | -0.064 |  |  |  |  |  |  |  |  |  |  |  |  |  |  |  |  |  |  |  |  |  |  |  |  |  |  |  |  |
| aSE2 | 0.131 | -0.032 |  |  |  |  |  |  |  |  |  |  |  |  |  |  |  |  |  |  |  |  |  |  |  |  |  |  |  |
| AP4 | 0.223 | -0.037 | 0.353 |  |  |  |  |  |  |  |  |  |  |  |  |  |  |  |  |  |  |  |  |  |  |  |  |  |  |
| RP3 | 0.355 | **0.078** | 0.275 | 0.390 |  |  |  |  |  |  |  |  |  |  |  |  |  |  |  |  |  |  |  |  |  |  |  |  |  |
| cSE2 | 0.269 | -0.128 | 0.454 | 0.451 | 0.475 |  |  |  |  |  |  |  |  |  |  |  |  |  |  |  |  |  |  |  |  |  |  |  |  |
| AP1 | 0.256 | 0.058 | 0.312 | **0.648** | 0.340 | 0.466 |  |  |  |  |  |  |  |  |  |  |  |  |  |  |  |  |  |  |  |  |  |  |  |
| aSE1 | 0.227 | -0.155 | **0.585** | 0.506 | 0.351 | 0.614 | 0.46 |  |  |  |  |  |  |  |  |  |  |  |  |  |  |  |  |  |  |  |  |  |  |
| cSE3 | 0.210 | -0.09 | 0.400 | 0.477 | 0.436 | **0.750** | 0.451 | 0.555 |  |  |  |  |  |  |  |  |  |  |  |  |  |  |  |  |  |  |  |  |  |
| CP4 | 0.236 | 0.057 | 0.253 | 0.422 | 0.267 | 0.425 | 0.592 | 0.349 | 0.502 |  |  |  |  |  |  |  |  |  |  |  |  |  |  |  |  |  |  |  |  |
| AC3 | 0.217 | 0.125 | 0.252 | 0.343 | 0.257 | 0.351 | 0.425 | 0.297 | 0.391 | 0.485 |  |  |  |  |  |  |  |  |  |  |  |  |  |  |  |  |  |  |  |
| OE3 | **0.481** | 0.152 | 0.195 | 0.306 | 0.347 | 0.307 | 0.380 | 0.232 | 0.273 | 0.399 | 0.369 |  |  |  |  |  |  |  |  |  |  |  |  |  |  |  |  |  |  |
| RP1 | 0.366 | -**0.07** | 0.305 | 0.303 | **0.572** | 0.485 | 0.280 | 0.360 | 0.371 | 0.222 | 0.178 | 0.307 |  |  |  |  |  |  |  |  |  |  |  |  |  |  |  |  |  |
| CP1 | 0.198 | 0.160 | 0.209 | 0.531 | 0.254 | 0.370 | 0.649 | 0.274 | 0.413 | **0.722** | 0.535 | 0.446 | 0.204 |  |  |  |  |  |  |  |  |  |  |  |  |  |  |  |  |
| AP3 | 0.274 | -0.033 | 0.351 | **0.590** | 0.347 | 0.464 | **0.727** | 0.500 | 0.459 | 0.567 | 0.470 | 0.320 | 0.315 | 0.583 |  |  |  |  |  |  |  |  |  |  |  |  |  |  |  |
| CP3 | 0.167 | 0.127 | 0.263 | 0.421 | 0.172 | 0.335 | 0.533 | 0.250 | 0.329 | **0.581** | 0.457 | 0.396 | 0.109 | 0.664 | **0.543** |  |  |  |  |  |  |  |  |  |  |  |  |  |  |
| AP2 | 0.184 | 0.016 | 0.327 | **0.559** | 0.334 | 0.396 | **0.673** | 0.407 | 0.420 | 0.567 | 0.453 | 0.360 | 0.248 | 0.587 | **0.721** | 0.488 |  |  |  |  |  |  |  |  |  |  |  |  |  |
| CP2 | 0.230 | 0.097 | 0.243 | 0.481 | 0.263 | 0.433 | 0.626 | 0.343 | 0.483 | **0.700** | 0.546 | 0.430 | 0.189 | 0.806 | 0.616 | **0.688** | 0.659 |  |  |  |  |  |  |  |  |  |  |  |  |
| OE2 | **0.573** | -0.040 | 0.304 | 0.310 | 0.402 | 0.349 | 0.272 | 0.318 | 0.307 | 0.258 | 0.257 | **0.495** | 0.501 | 0.226 | 0.202 | 0.210 | 0.194 | 0.255 |  |  |  |  |  |  |  |  |  |  |  |
| INT2 | 0.270 | -0.008 | 0.406 | 0.369 | 0.345 | 0.438 | 0.331 | 0.507 | 0.396 | 0.311 | 0.331 | 0.364 | 0.450 | 0.258 | 0.38 | 0.227 | 0.317 | 0.227 | 0.392 |  |  |  |  |  |  |  |  |  |  |
| AC2 | 0.192 | 0.016 | 0.328 | 0.266 | 0.145 | 0.337 | 0.355 | 0.385 | 0.338 | 0.371 | **0.343** | 0.204 | 0.256 | 0.328 | 0.361 | 0.229 | 0.301 | 0.386 | 0.226 | 0.399 |  |  |  |  |  |  |  |  |  |
| RP2 | 0.390 | **0.047** | 0.316 | 0.389 | **0.508** | 0.520 | 0.357 | 0.352 | 0.444 | 0.358 | 0.265 | 0.435 | **0.572** | 0.369 | 0.377 | 0.288 | 0.296 | 0.369 | 0.507 | 0.400 | 0.325 |  |  |  |  |  |  |  |  |
| cSE1 | 0.184 | -0.100 | 0.506 | 0.485 | 0.451 | **0.722** | 0.490 | 0.613 | **0.663** | 0.477 | 0.337 | 0.275 | 0.412 | 0.393 | 0.474 | 0.355 | 0.459 | 0.419 | 0.335 | 0.468 | 0.351 | 0.517 |  |  |  |  |  |  |  |
| cSE4 | 0.202 | -0.096 | 0.430 | 0.386 | 0.382 | **0.647** | 0.408 | 0.530 | **0.614** | 0.472 | 0.374 | 0.312 | 0.435 | 0.398 | 0.436 | 0.361 | 0.405 | 0.423 | 0.406 | 0.475 | 0.372 | 0.502 | **0.766** |  |  |  |  |  |  |
| RP4 | 0.288 | **-0.039** | 0.386 | 0.363 | **0.442** | 0.452 | 0.378 | 0.444 | 0.523 | 0.448 | 0.372 | 0.381 | **0.490** | 0.406 | 0.428 | 0.317 | 0.311 | 0.404 | 0.437 | 0.429 | 0.250 | **0.570** | 0.488 | 0.570 |  |  |  |  |  |
| INT1 | 0.071 | -0.065 | 0.258 | 0.105 | 0.029 | 0.146 | 0.111 | 0.242 | 0.079 | 0.131 | 0.060 | 0.148 | 0.048 | 0.128 | 0.153 | 0.164 | 0.125 | 0.187 | 0.182 | **0.130** | 0.211 | 0.162 | 0.166 | 0.235 | 0.235 |  |  |  |  |
| AC1 | 0.288 | -0.055 | 0.379 | 0.323 | 0.327 | 0.408 | 0.404 | 0.495 | 0.377 | 0.375 | **0.311** | 0.294 | 0.353 | 0.356 | 0.444 | 0.268 | 0.377 | 0.378 | 0.270 | 0.464 | **0.532** | 0.380 | 0.491 | 0.436 | 0.382 | 0.349 |  |  |  |
| aSE3 | 0.254 | -0.173 | **0.534** | 0.381 | 0.339 | 0.517 | 0.448 | **0.617** | 0.458 | 0.432 | 0.307 | 0.282 | 0.370 | 0.360 | 0.489 | 0.330 | 0.433 | 0.381 | 0.293 | 0.523 | 0.351 | 0.358 | 0.566 | 0.533 | 0.522 | 0.257 | 0.624 |  |  |
| RP5 | 0.268 | -**0.101** | 0.466 | 0.326 | **0.428** | 0.461 | 0.386 | 0.399 | 0.422 | 0.328 | 0.314 | 0.308 | **0.507** | 0.276 | 0.401 | 0.230 | 0.375 | 0.307 | 0.450 | 0.445 | 0.398 | **0.547** | 0.492 | **0.526** | 0.494 | 0.237 | 0.456 | 0.506 |  |

*Note*: Correlation coefficients in bold are per subscale
